# A double-blind randomized trial between risperidone and haloperidol in drug-naive patients with paranoid schizophrenia

**DOI:** 10.4103/0019-5545.46071

**Published:** 2005

**Authors:** K.J. Vijay Sagar, C.R. Chandrashekar

**Affiliations:** * Assistant Professor, Department of Psychiatry, S.V. Medical College, Tirupati 517507, Tamil Nadu; ** Professor, NIMHANS, Bangalore

**Keywords:** Risperidone, haloperidol, drug-naive, efficacy, adverse effects

## Abstract

**Background:**

Haloperidol is highly effective against the positive symptoms of schizophrenia, but is less effective against the negative symptoms. It also produces marked extrapyramidal symptoms. The newer atypical antipsychotics are believed to have an equal efficacy with a favourable side-effect profile.

**Aim:**

We assessed the effects of risperidone and haloperidol in patients with schizophrenia to compare their clinical efficacy and side-effect profile.

**Methods:**

A double-blind, randomized, prospective, comparative trial with a parallel treatment design of 6 weeks' duration was undertaken on 46 drug-naive schizophrenics to compare the efficacy and adverse effects profile of risperidone and haloperidol.

**Results:**

The study showed no difference in the positive and negative subscale scales on the Positive and Negative Syndrome Scale (PANSS). However, risperidone was found to have an edge over haloperidol in improving the general psychopathology as well as in bringing about global improvement.

**Conclusion:**

Of the two drugs, the adverse effect profile of risperidone is better, with less need for the use of antiparkinsonian medication.

## INTRODUCTION

Approximately 50% of patients with schizophrenia do not obtain adequate relief with conventional neuroleptics.^1^ Though haloperidol is highly effective against the hallu-cinations and delusions of schizophrenia, it is not as useful in controlling the negative symptoms. Moreover, the potent dopamine antagonism exhibited by haloperidol is associated with a high rate of occurrence of extrapyramidal symptoms (EPS). The development of a new class of antipsychotic drugs that bind both serotonin (5-hydroxytryptamine [5-HT_2_]) and dopamine (D2) receptors is a major advancement. In this class, risperidone favourably evokes a lower incidence of concurrent EPS.

During the past decade, a number of double-blind trials have compared the effect of risperidone with that of haloperidol to prove its supremacy along with its favourable adverse effect profile.^2^–^5^ However, certain studies showed equal efficacy.^6^–^10^ It is noteworthy that among these, only about 4 studies^11^–^14^ were on drug-naive patients and only one was a double-blind trial. Moreover, only a few studies were done on the Indian population and this prompted the author to take up this study to evaluate the comparative efficacy and tolerability of risperidone and haloperidol in the treatment of drug-naive schizophrenics.

## METHODS

The sample (*n*=46) was selected from the outpatient department of the National Institute of Mental Health and Neuro Sciences (NIMHANS), Bangalore using the criteria described below.

### Inclusion criteria

1. The age of the subjects (males and females) was in the range of 18–45 years.
2. All subjects had paranoid schizophrenia.
3. The subjects should not have received any antipsychotic drug.
4. Informed consent was taken from the patient and/or a family member.

### Exclusion criteria

Patients with the following were excluded:

1. Co-morbid substance dependence, mood disorder, personality disorders
2. Evidence of organic conditions such as dementia and epilepsy.

Except for the level of education, the two groups did not differ on any of the other sociodemographic variables such as age, sex, place of origin, marital status, occupation, duration of illness and family history.

The selected patients were admitted and randomly grouped (23 each) to receive risperidone or haloperidol therapy and the investigator was kept blind to the assignment. At baseline, along with a complete psychiatric history and physical examination, assessment in both the groups was done using the Positive and Negative Syndrome Scale (PANSS)^15^ and the Clinical Global Improvement (CGI) scale^16^ for efficacy, and the Udvalg for Kliniske Undersogelser (UKU) side-effect rating scale for tolerability.^17^ The initial daily doses of risperidone and haloperidol were 2 mg and 5 mg, respectively, which were subsequently increased as per the need, reaching a maximum daily dose of 8 mg for risperidone and 15 mg for haloperidol at the end-point.

The patients were assessed at weekly intervals for 6 weeks using PANSS, which was the key measure of antipsychotic efficacy. The primary measure of efficacy was the percentage of patients showing clinical improvement defined a priori as 20% reduction from the baseline in the total PANSS score at the end-point. The CGI scale was used to evaluate the overall status at week 3 and week 6.

The patients were also assessed every week till the end-point using the UKU side-effect rating scale. While no other antipsychotic treatment was allowed, EPS in both the groups were treated with the antiparkinsonian drug trihexyphenidyl, as per the need.

For analysis, parametric statistical methods such as ANOVA and *t* test were applied. Non-parametric statistical tests in the form of the chi-square test was also applied. For the quality of variance, Levene test was used.

## RESULTS

### Efficacy parameters

#### Efficacy on PANSS

There was no difference between the two drugs in the improvement of positive and negative symptoms (Table 1).

**Table 1:** Mean (±SD) scores on the PANSS and the CGI scale at baseline and end-point (risperidone vs haloperidol)

	Risperidone (*n*=23)	Haloperidol (*n*=23)
Positive PANSS		
Baseline	15.13 ± 4.04	15.70 ± 2.88
End-point	6.70 ± 2.34*	8.17 ± 2.29
Negative PANSS		
Baseline	17.39 ± 4.70	15.61 ± 5.01
End-point	10.61 ± 3.85*	12.00 ± 4.09
General psychopathology		
Baseline	33.30 ± 5.79	32.43 ± 6.19
End-point	16.65 ± 6.60**	33.13 ± 6.08
CGI severity		
Baseline	4.35 ± 0.49	4.39 ± 0.50
End-point	2.83 ± 0.49**	3.30 ± 0.56

PANSS: Positive and Negative Syndrome Scale; CGI: Clinical Global Improvement

* p>0.05 (not statistically significant);

** p<0.05 (statistically significant)

#### Efficacy on the CGI Scale

Significant global improvement (p=0.05) and reduction in severity (p=0.023) were noted (Table 1).

### Adverse effects

Application of RMANOVA showed that significant differences exist between the groups with regard to sedation, increased duration of sleep, tremor, constipation, polyuria/polydipsia and weight gain (Table 2).

**Table 2:** Significant side-effects on the Udvalg for Kliniske Undersogelser (UKU) side-effect rating scale

	Between weeks			Between groups		
		
Side-effects	F	df	p	F	df	p
Sedation	3.084	6,264	0.006	6.835	1,44	0.012
Increased duration of sleep	2.784	6,264	0.012	5.989	1,44	0.0018
Tremor	17.498	6,264	0	5.434	1,44	0.024
Constipation	10.463	6,264	0	9.406	1,44	0.004
Polyuria/polydipsia	9.094	6,264	0	4.982	1,44	0.031
Weight gain	1.436	6,264	0.201	6.154	1,44	0.017

df: degrees of freedom

Patients in both the groups needed antiparkinsonian medication (trihexyphenidyl)—12 patients in the risperidone group and 15 patients in the haloperidol group. The average dose used was 1.39 mg for the risperidone group and 1.7 mg for the haloperidol group (Table 3).

**Table 3:** Requirement of antiparkinsonian medication (trihexyphenidyl) (risperidone vs haloperidol)

Antiparkinsonian medication (trihexyphenidyl)	Risperidone group (*n*=23)	Haloperidol group (*n*=23)	Chisquare test	Significance
Not received	11 (47.8)	8 (34.8)	0.93	Not significant
Received	12 (52.2)	15 (65.2)		

Values in parentheses are percentages.

## DISCUSSION

In this randomized, double-blind, 6-week study, though marked improvement of 56% vs 48% on the positive subscale and 39% vs 23% on the negative subscale of PANSS was recorded, for risperidone and haloperidol, respectively, there was no statistical difference between the two groups. Thus, our conclusion of equal efficacy is in concurrence with the results of others studies.^6^,^7^,^9^,^10^ Better efficacy with risperidone was recorded by some authors.^3^–^5^,^11^,^12^,^18^,^19^ However, in the general psychopathology subscale of PANSS and in terms of severity and global improvement on the CGI scale, risperidone showed more efficacy than haloperidol.

The majority of studies reporting otherwise, i.e. showing better efficacy of risperidone were on patients with chronic schizophrenia where negative symptoms are said to be prominent; the patients had responded more favourably with risperidone, which is known for its hallmark effect on negative symptoms.^3^,^4^ Some noteworthy features of these studies include large sample sizes, i.e. 1362, 523 and 183, respectively and longer duration of trial, i.e. 8,12 and 16 weeks, respectively.

As in many other studies,^3^–^5^,^12^,^20^ the present study also showed a lower incidence and severity of adverse effects. Kontaxakis *et al*. during their study on 17 drug-naive, first- episode schizophrenic patients showed that all the patients reached the optimal dose of risperidone before developing EPS.^14^

The present study also evaluated the need for antiparkin-sonian medication and showed that although the risperidone group needed less therapeutic intervention, it was not statistically significant between the groups. This finding concurred with the lone multicentric study by Agarwal *et al*.^21^

The merits of this study are its double-blind design, use of a flexible dosage schedule and good drug compliance. The limitations of this study include the small sample size and short duration of follow-up.
